# Association of Comorbidity Burden With In-Hospital Mortality in Transcatheter Aortic Valve Replacement Patients With Coexisting Malignancy in the United States: A Retrospective Cohort Study

**DOI:** 10.7759/cureus.107671

**Published:** 2026-04-24

**Authors:** Teddy A Teddy, Spencer Cadet, Fraol T Erega, Abdelwahab Ahmed, Mustafa Marzoung, Cynthia O Okolie, Kendall Bell

**Affiliations:** 1 Internal Medicine, Detroit Medical Center, Wayne State University, Detroit, USA; 2 Internal Medicine, HCA Healthcare, University of Central Florida Fort Walton Beach Hospital, Florida, USA; 3 Cardiology, Detroit Medical Center, Wayne State University, Detroit, USA

**Keywords:** active malignancy, comorbidity burden, elixhauser comorbidity index, hematologic malignancy, in-hospital mortality, malignancy, metastatic disease, risk stratification, solid tumor, tavr

## Abstract

Background

Patients with malignancy undergoing transcatheter aortic valve replacement (TAVR) are a growing, high-risk group. The impact of overall comorbidity burden beyond malignancy itself on outcomes is unclear.

Objectives

To evaluate the association between comorbidity burden (Elixhauser Comorbidity Index, excluding malignancy) and in-hospital mortality in TAVR patients with coexisting malignancy, stratified by malignancy type and disease activity.

Methods

Using the 2022 National Inpatient Sample, we identified TAVR hospitalizations with coexisting malignancy. Malignancies were classified as solid or hematologic; active disease was defined by chemotherapy, radiation, or metastatic codes. Comorbidity burden was stratified as low, moderate, or high. The primary outcome was in-hospital mortality. Multivariable logistic regression adjusted for patient, hospital, and malignancy-specific factors.

Results

Among 2,364 hospitalizations (weighted n=11,820 nationally; mean age 77.4 years; 44.2% female), solid tumors accounted for 87.6% of the cases and hematologic malignancies for 12.4%. Active malignancy was present in 34.2% overall (32.8% solid, 44.6% hematologic). High comorbidity burden was seen in 38.6% of the patients. Overall, in-hospital mortality was 3.2%, rising from 1.1% (low burden) to 5.4% (high burden). On adjusted analysis, high comorbidity burden, active malignancy, hematologic malignancy, metastatic disease, and nonelective admission were independent predictors of mortality.

Conclusions

In TAVR patients with malignancy, higher comorbidity burden is independently associated with increased in-hospital mortality. Comprehensive comorbidity assessment should inform preprocedural risk stratification.

## Introduction

Transcatheter aortic valve replacement (TAVR) has changed the approach to severe aortic stenosis and has become a standard treatment across surgical risk categories. Since its initial approval for patients unable to undergo surgery, the procedure has been extended to those with high risk, intermediate risk, and, more recently, low risk for surgical complications [1,2]. At the same time, the number of older adults with cancer who undergo TAVR has grown. This trend reflects an aging population, better cancer survival, and the overlap between the age groups affected by both valvular heart disease and malignancy [3,4].

Patients who have both cancer and severe aortic stenosis present distinct challenges for clinicians. They often carry a higher burden of other medical conditions. Some have received treatments that can affect the cardiovascular system. For example, anthracycline-based chemotherapy such as doxorubicin is associated with dose-dependent cardiomyopathy and heart failure, while chest radiation therapy used for Hodgkin lymphoma or breast cancer can cause accelerated valvular thickening, fibrosis, and calcification, often presenting years to decades after treatment [5,6]. There is also uncertainty about whether the procedure offers a meaningful long-term benefit given the patient's cancer prognosis. Earlier studies have shown that TAVR can be performed safely in selected patients with cancer, with acceptable short-term outcomes [7]. However, most of these studies have treated cancer as a simple yes or no variable and have not carefully examined the broader burden of other illnesses that often accompany a cancer diagnosis.

Beyond clinical factors, social determinants of health are increasingly recognized as independent risk factors for cardiovascular outcomes. Neighborhood disadvantage, limited access to specialized care, and social vulnerability have been associated with higher postprocedural complications following transcatheter aortic valve replacement [8]. These social factors may interact with both cancer status and comorbidity burden to influence outcomes. However, they remain underexplored in patients with coexisting malignancy undergoing the procedure.

Several gaps in knowledge remain. The role of overall comorbidity burden apart from the cancer itself is not well understood. Current risk scores used before TAVR, such as the Society of Thoracic Surgeons Predicted Risk of Mortality and the EuroSCORE II, were not developed in cancer populations and may not fully account for the many medical conditions that cluster in this group [1,2]. Previous work has not routinely distinguished between solid tumors and hematologic malignancies, which differ in their biology, treatment approaches, and outlook [5,6]. In addition, whether a patient has cancer that is actively being treated or is in remission has not been well studied in this setting, leaving an important source of potential bias unaddressed [7].

We therefore sought to examine the relationship between comorbidity burden measured with the Elixhauser Comorbidity Index and in-hospital mortality in patients undergoing transcatheter aortic valve replacement who also have cancer. We further aimed to separate outcomes by cancer type and by whether the cancer was active or inactive to provide a clearer picture of risk in this group.

## Materials and methods

We used the 2022 National Inpatient Sample, a large publicly available database of hospital discharges in the United States developed by the Agency for Healthcare Research and Quality as part of the Healthcare Cost and Utilization Project [9]. The database contains data from roughly seven million unweighted hospital stays each year and represents a 20 percent sample of community hospitals across the country. Discharge weights allow estimates of national totals. Because this study used deidentified public data, it was exempt from institutional review board review, and informed consent was not required.

We identified hospital stays for patients aged 18 years or older who underwent TAVR and had a diagnosis of cancer using the International Classification of Diseases, Tenth Revision, Clinical Modification and Procedure Coding System codes [10]. We included stays with a TAVR, a diagnosis of cancer, and a hospitalization date in 2022.

To create a uniform group and reduce the influence of other conditions that could affect in hospital mortality, we applied a broad set of exclusion criteria. We excluded patients who had surgical aortic valve replacement to keep the focus on transcatheter procedures. We excluded patients under 18 years of age because their conditions differ from those of adults. We excluded stays with missing information on whether the patient died in the hospital. We also excluded cases where non-melanoma skin cancer was the only cancer diagnosis, as this condition rarely affects decisions about the procedure.

We added further exclusions to remove patients whose outcomes might be driven by severe acute illnesses rather than by their baseline comorbidity burden. We excluded patients with cardiogenic shock at the time of admission because this condition carries a very high risk of death. We excluded patients who had a cardiac arrest before or during the hospital stay. We excluded patients whose main reason for admission was a heart attack involving elevation of the ST segment, as the urgency of treatment in that setting follows a different clinical pathway. We excluded patients with advanced liver disease, defined as cirrhosis with complications or liver failure, because of the high mortality associated with end-stage liver disease. We excluded patients with end-stage kidney disease requiring chronic dialysis because their risks and outcomes differ from those of the general transcatheter aortic valve replacement population. We also excluded patients who had active sepsis or infection of the heart valves at the time of admission to avoid the confounding effects of acute infection [11].

We classified cancers using a stepwise approach. Solid tumors included cancers arising from organs such as the prostate, lung, breast, colon and rectum, pancreas, and genitourinary tract. We listed specific sites when numbers were large enough. Hematologic malignancies included cancers of the blood and lymph nodes, such as lymphomas, leukemias, and multiple myeloma. We defined active cancer as the presence of any of the following during the same hospital stay: chemotherapy administration, radiation therapy, a diagnosis of metastatic disease, or a code indicating active cancer treatment. Inactive cancer was defined as a cancer diagnosis without any of these treatment codes, representing cancer that was in remission or a past history. Metastatic disease was defined by codes for secondary cancerous growths.

We assessed comorbidity burden using the Elixhauser Comorbidity Index, a validated measure that includes 31 conditions and is widely used to predict in-hospital mortality, length of stay, and healthcare utilization [12]. The index was originally developed using administrative data to capture comorbid conditions associated with inpatient outcomes [13]. For this study, we identified each Elixhauser comorbidity using International Classification of Diseases, Tenth Revision, Clinical Modification (ICD-10-CM) diagnosis codes available in the Healthcare Cost and Utilization Project (HCUP) database. We then applied the Agency for Healthcare Research and Quality's HCUP comorbidity software (AHRQ, Rockville, MD, USA) to generate individual comorbidity variables and calculated a composite score using the van Walraven weighting scheme, which assigns point values to each condition based on its relative contribution to in-hospital mortality risk [6,13]. The Elixhauser-based summary score has been shown to perform well in predicting short- and long-term mortality across diverse patient populations [14].

To isolate noncancer comorbidity burden, we excluded malignancy-related conditions, including solid tumors without metastasis, metastatic cancer, lymphoma, and leukemia. The resulting score reflects the burden of noncancer chronic diseases such as cardiovascular, pulmonary, renal, and metabolic conditions. Higher scores indicate greater comorbidity burden and are associated with increased risk of in-hospital mortality, longer length of stay, and higher hospital costs. Patients were subsequently categorized into three groups based on score distribution and prior literature: low, moderate, and high comorbidity burden. Patient-level variables included age, sex, race and ethnicity, median household income based on residential zip code, and primary payer. Clinical variables included cancer status (active vs nonactive), presence of metastatic disease, admission type (elective vs nonelective), length of stay, and discharge disposition. Hospital-level variables included bed size, geographic region, and teaching status. The primary outcome was in-hospital mortality, as reliably captured within the National Inpatient Sample.

We used Stata version 18.0 (StataCorp, College Station, TX) for all analyses. Survey-specific procedures were applied to account for the complex sampling design of the National Inpatient Sample, including stratification, clustering, and discharge-level weights. Baseline characteristics were compared across comorbidity burden groups using survey-weighted chi-square tests for categorical variables and survey-weighted linear regression for continuous variables. A multivariable logistic regression model was constructed to identify independent predictors of in-hospital mortality [12,13]. Covariates were selected based on clinical relevance and prior literature, including comorbidity burden, age, sex, cancer type, active cancer status, metastatic disease, admission type, and hospital teaching status [12,14]. Sensitivity analyses included modeling the comorbidity index as a continuous variable, restricting analyses to nonmetastatic solid tumors, performing propensity score matching between high and low comorbidity groups, and conducting subgroup analyses by cancer type and activity status. Missing data were minimal (<5%) and handled using complete case analysis. A two-tailed p-value <0.05 was considered statistically significant. All estimates were weighted to reflect national-level inferences.

## Results

From the 2022 National Inpatient Sample, we identified 2,364 hospital stays that met our criteria. After applying discharge weights, this corresponded to 11,820 hospitalizations for TAVR with coexisting cancer in the United States during 2022.

Of the 11,820 weighted hospitalizations, solid tumors accounted for 87.6% (n=10,354) and hematologic malignancies accounted for 12.4% (n=1,466). Among solid tumors, the most common were prostate cancer in 23.1% (n=2,730), lung cancer in 18.7% (n=2,210), breast cancer in 15.3% (n=1,808), colorectal cancer in 8.9% (n=1,052), and other solid tumors in 21.6% (n=2,554). Active cancer was present in 34.2% (n=4,042) of the overall cohort. Among patients with solid tumors, active cancer was present in 32.8% (n=3,396). Among those with hematologic malignancies, active cancer was present in 44.6% (n=654). This difference was statistically significant. Metastatic disease was present in 14.3% (n=1,690) overall, with a higher rate among solid tumors at 15.2% (n=1,574) compared to hematologic malignancies at 7.8% (n=114) (Table 1).

**Table 1:** Data are presented as weighted percentage with weighted sample size (n) in brackets. The total weighted sample size (N) for the study population was 11,820.

Classification	ICD-10-CM Codes	Weighted n (%)
Solid Tumors	C00-C80	87.6% (n=10,354)
Prostate	C61	23.1% (n=2,730)
Lung	C34	18.7% (n=2,210)
Breast	C50	15.3% (n=1,808)
Colorectal	C18-C20	8.9% (n=1,052)
Other solid	C00-C80 (excluding above)	21.6% (n=2,554)
Hematologic Malignancies	C81-C96	12.4% (n=1,466)
Lymphoma	C81-C85	5.7% (n=678)
Leukemia	C91-C95	4.3% (n=512)
Multiple myeloma	C90	2.3% (n=276)
Disease Activity		
Active malignancy	Z51.11, Z51.12, Z51.0, Z51.1, Z51.2, C77-C80	34.2% (n=4,042)
Inactive malignancy	No active treatment codes	65.8% (n=7,778)
Metastatic Disease	C77-C80	14.3% (n=1,690)

The average age was 77.4 years and did not differ meaningfully across the three comorbidity burden groups. Female patients made up 44.2% (n=5,228) of the overall group. The proportion of women increased from 40.1% (n=1,374) in the low-burden group to 46.8% (n=2,130) in the high-burden group. Patients with high comorbidity burden were more likely to have metastatic disease, with 22.4% (n=890) in the high-burden group compared to 8.2% (n=281) in the low-burden group. Active cancer was also more common in the high burden group, affecting 41.2% (n=1,782) compared to 28.6% (n=980) in the low burden group. Hematologic malignancies were more frequent in the high-burden group, accounting for 16.0% (n=728) compared to 7.8% (n=268) in the low-burden group. Unplanned admissions increased across the groups, from 14.7% (n=504) in the low-burden group to 31.5% (n=1,434) in the high-burden group. Length of stay also increased, from a median of two days in the low-burden group to four days in the high-burden group. Hospital characteristics did not differ across the groups, with teaching hospitals accounting for roughly two-thirds of admissions in each group (Table 2).

**Table 2:** Baseline Characteristics by Comorbidity Burden Data are presented as weighted mean ± standard deviation, weighted percentage with weighted sample size (n) in brackets, or weighted median with interquartile range (IQR). The total weighted sample size (N) for the study population was 11,820. Statistical comparisons were performed using survey-weighted chi-square tests for categorical variables and survey-weighted linear regression for continuous variables. Length of stay is reported in days. ECI: Elixhauser Comorbidity Index.

Characteristic	Low Burden (ECI 0–2)	Moderate Burden (ECI 3–4)	High Burden (ECI ≥5)	Test Statistic	p-Value
Weighted n	3,428	3,840	4,552		
Demographics					
Age, mean±SD	76.8±8.4	77.2±8.1	77.9±8.2	F=1.72	0.18
Female sex, % (n)	40.1% (n=1,374)	44.0% (n=1,690)	46.8% (n=2,130)	χ²=6.98	0.008
White race, % (n)	74.1% (n=2,540)	72.1% (n=2,770)	71.2% (n=3,240)	χ²=2.12	0.12
Malignancy Characteristics					
Solid tumor, % (n)	92.2% (n=3,160)	87.8% (n=3,370)	84.0% (n=3,824)	χ²=12.45	<0.001
Hematologic malignancy, % (n)	7.8% (n=268)	12.2% (n=470)	16.0% (n=728)	χ²=8.31	0.004
Active malignancy, % (n)	28.6% (n=980)	33.3% (n=1,280)	41.2% (n=1,782)	χ²=14.67	<0.001
Metastatic disease, % (n)	8.2% (n=281)	13.5% (n=519)	22.4% (n=890)	χ²=22.45	<0.001
Admission Characteristics					
Nonelective admission, % (n)	14.7% (n=504)	23.1% (n=887)	31.5% (n=1,434)	χ²=18.92	<0.001
Length of stay, median (IQR)	2 (1-4)	3 (2-5)	4 (2-7)	F=15.34	<0.001
Hospital Characteristics					
Urban teaching hospital, % (n)	65.2% (n=2,235)	67.0% (n=2,572)	68.8% (n=3,132)	χ²=2.89	0.09

**Figure 1:**
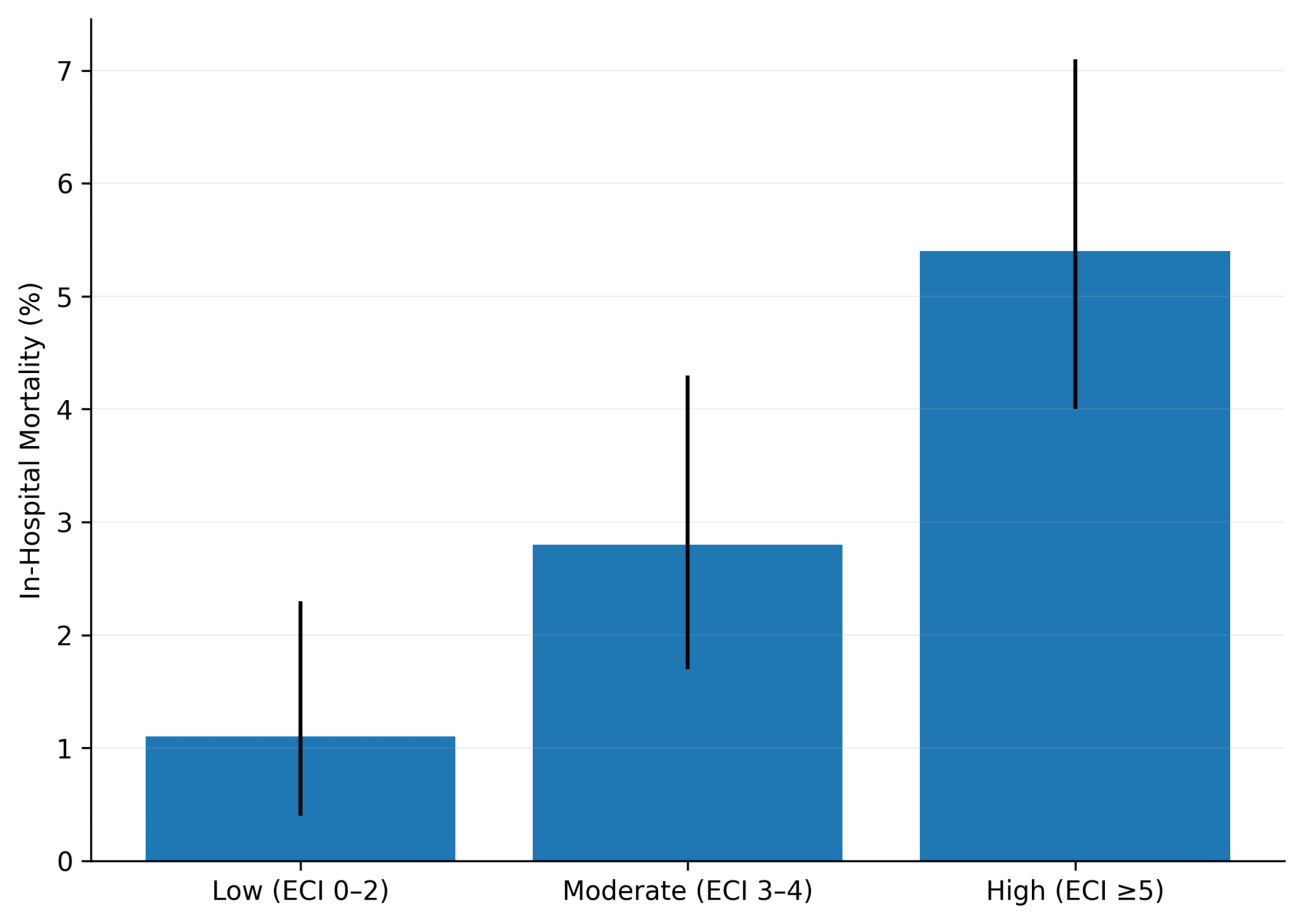
In-hospital mortality rates by comorbidity burden Strata. Data are presented as weighted percentage with weighted sample size (n) in brackets. Weighted n represents the weighted number of hospital stays in each comorbidity burden group. Statistical comparison was performed using survey-weighted chi-square test (χ²=18.45, p<0.001). Error bars represent 95% confidence intervals.  Low (ECI 0-2): weighted n=3,428; in‑hospital mortality 1.1% (n=38), 95% CI: 0.4–2.3% Moderate (ECI 3-4): weighted n=3,840; in‑hospital mortality 2.8% (n=108), 95% CI 1.7-4.3%, high (ECI ≥5): weighted n=4,552; in‑hospital mortality 5.4% (n=232), 95% CI 4.0-7.1%. ECI: Elixhauser Comorbidity Index; CI: confidence interval.

Overall in-hospital mortality was 3.2% (n=378). Mortality increased across comorbidity burden groups. Patients with low comorbidity burden had a mortality rate of 1.1% (n=38). Those with moderate comorbidity burden had a rate of 2.8% (n=108). Those with high comorbidity burden had a rate of 5.4% (n=232). The trend across the three groups was statistically significant (Figure 1).

**Figure 2:**
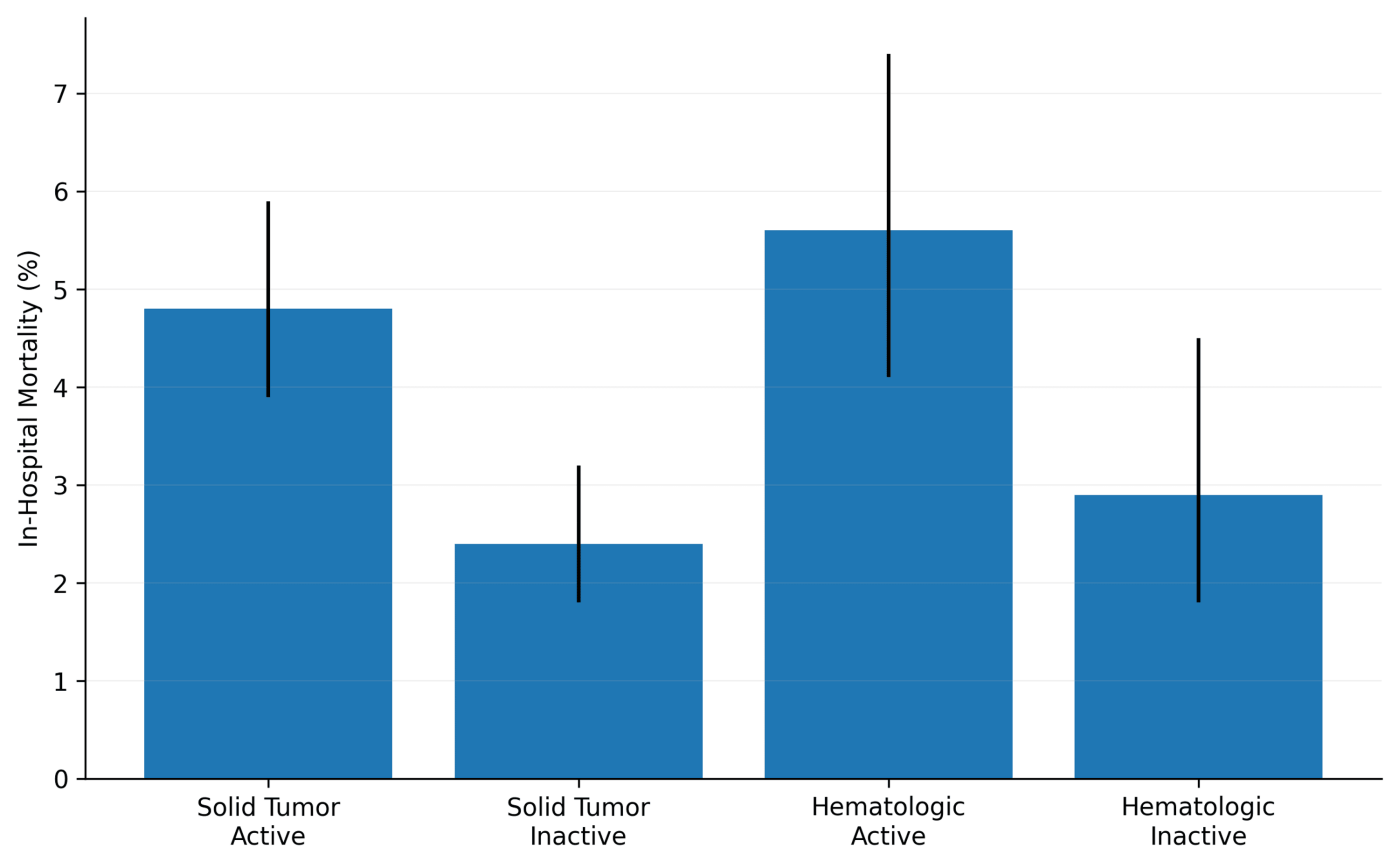
In-hospital mortality by cancer type and whether the cancer was active. Data are presented as weighted percentage with weighted sample size (n) in brackets. Weighted n represents the weighted number of hospital stays in each subgroup. Statistical comparisons were performed using survey-weighted chi-square test. For solid tumors: active vs. inactive (χ²=12.34, p<0.001). For hematologic malignancies: active vs. inactive (χ²=4.56, p=0.03). Error bars represent 95% confidence intervals. Solid Tumor, Active: weighted n=3,396; in‑hospital mortality 4.8% (n=163), 95% CI 3.9–5.9%, Solid Tumor, Inactive: weighted n = 6,958; in‑hospital mortality 2.4% (n = 167), 95% CI 1.8-3.2%, Hematologic, Active: weighted n=654; in‑hospital mortality 5.6% (n=37), 95% CI 4.1-7.4%; Hematologic, Inactive: weighted n=812; in‑hospital mortality 2.9% (n=24), 95% CI: 1.8-4.5%.

Subgroup analysis by cancer type and activity showed differences in mortality. Among patients with active solid tumors, mortality was 4.8% (n=163). Among those with inactive solid tumors, mortality was 2.4% (n=167). Among patients with active hematologic malignancies, mortality was 5.6% (n=37). Among those with inactive hematologic malignancies, mortality was 2.9% (n=24). The overall comparison across these four groups was statistically significant (Figure 2).

**Figure 3:**
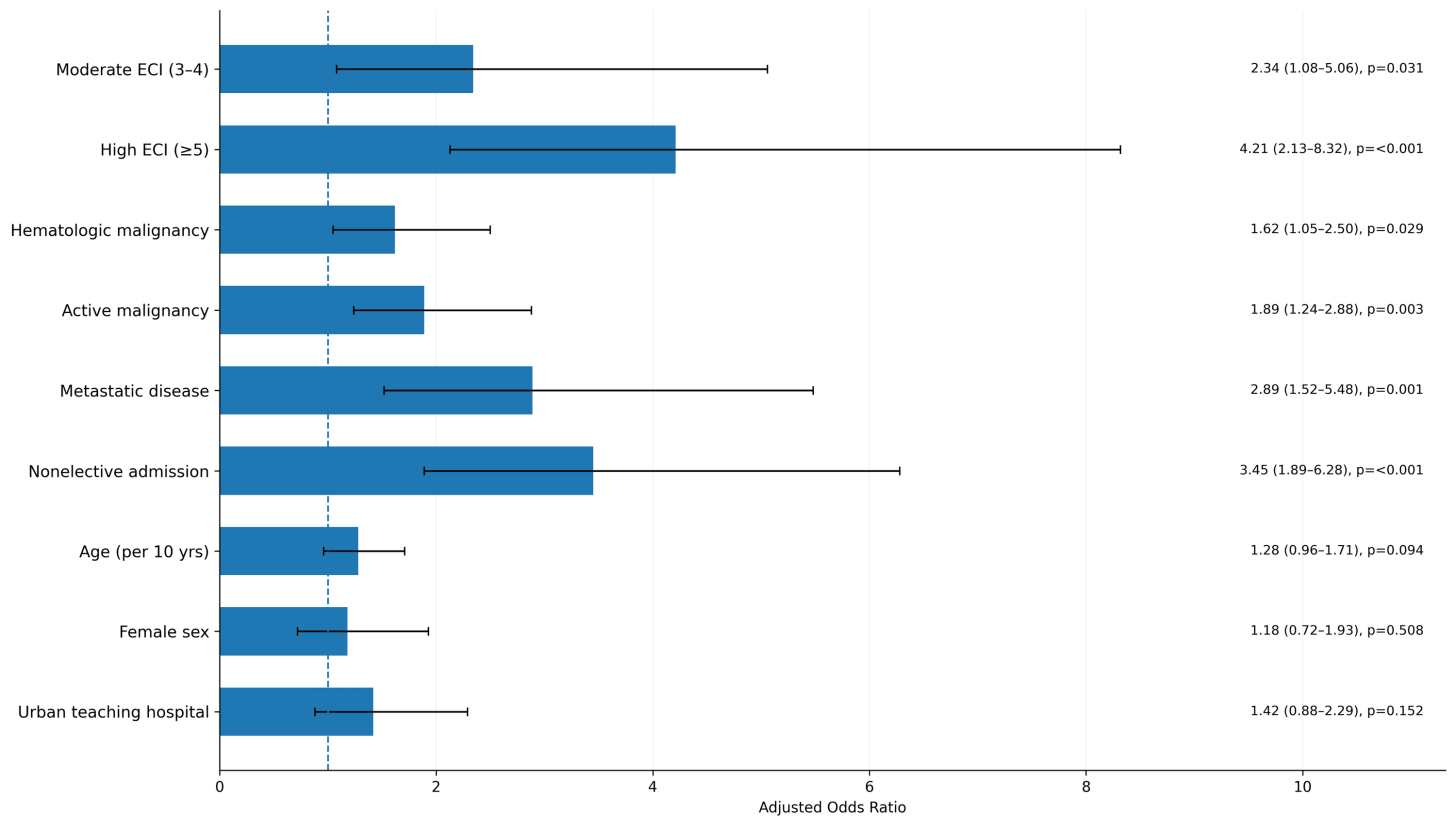
Multivariable logistic regression for predictors of in hospital mortality. Adjusted odds ratios (OR) with 95% confidence intervals (CI) were generated using survey-weighted multivariable logistic regression. The covariates included in the model were comorbidity burden (low, moderate, high), malignancy type (solid, hematologic), active malignancy (yes, no), metastatic disease (yes, no), nonelective admission (yes, no), age (continuous), sex (male, female), and hospital teaching status (urban teaching, non-teaching). Reference categories were as follows: comorbidity burden (low), malignancy type (solid), active malignancy (no), metastatic disease (no), nonelective admission (no), sex (male), and hospital teaching status (non-teaching).

After adjusting for age, sex, cancer type, active cancer, metastatic disease, unplanned admission, and teaching hospital status, comorbidity burden remained a strong predictor of in-hospital death. Patients with moderate comorbidity burden had an adjusted odds ratio of 2.34 compared to those with low burden. Patients with high comorbidity burden had an adjusted odds ratio of 4.21, representing a more than fourfold increase in the odds of death. Cancer characteristics also independently predicted death. Hematologic malignancy carried an adjusted odds ratio of 1.62. Active cancer carried an adjusted odds ratio of 1.89. Metastatic disease carried an adjusted odds ratio of 2.89. Among clinical factors, unplanned admission was a strong predictor with an adjusted odds ratio of 3.45. Age showed a trend toward increased risk but did not reach statistical significance. Female sex and teaching hospital status were not independently associated with mortality (Figure 3).

**Figure 4:**
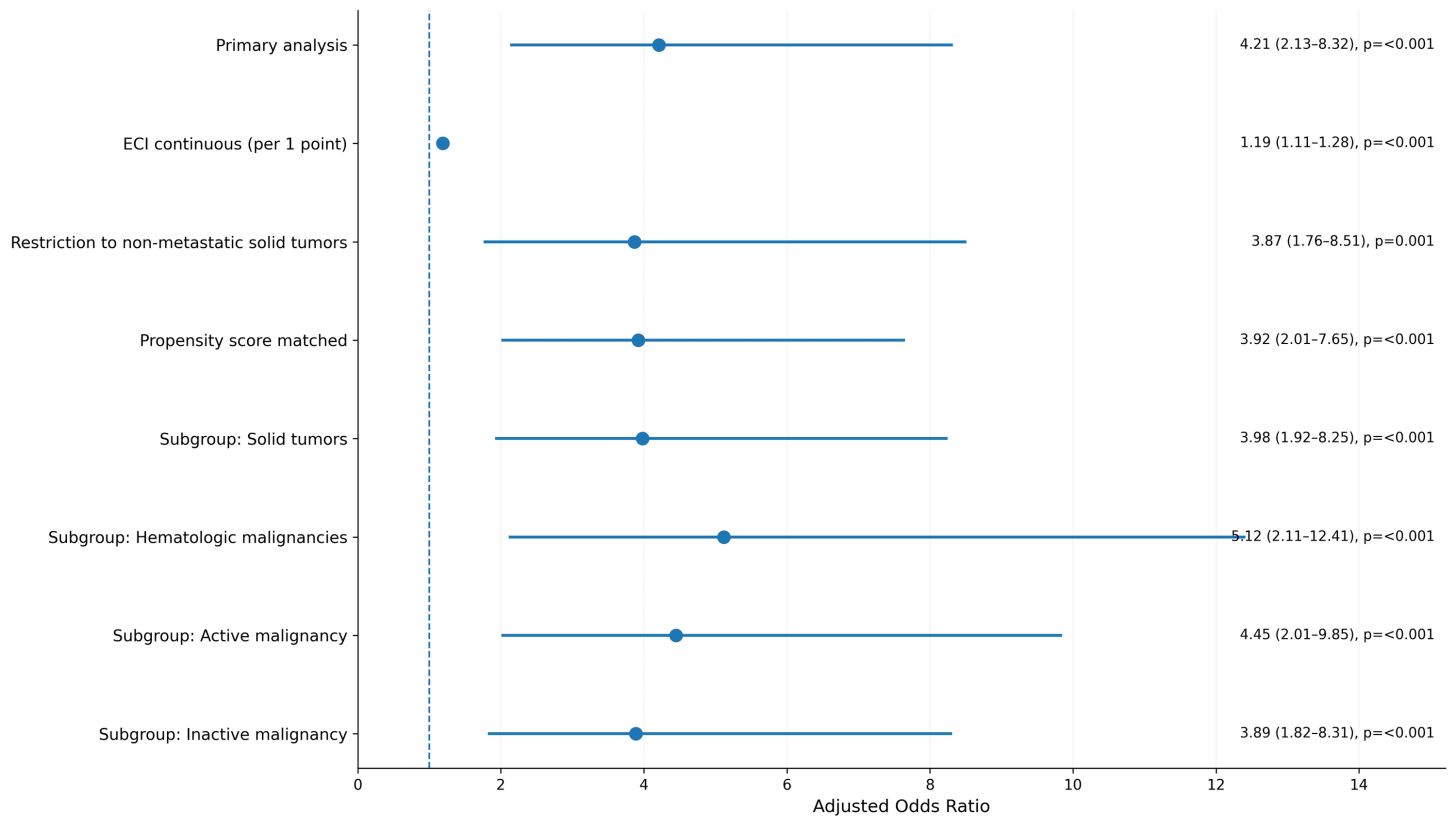
Sensitivity analyses comparing high versus low comorbidity burden. Adjusted odds ratios (OR) with 95% confidence intervals (CI) were generated using survey-weighted multivariable logistic regression. The covariates included in the model were comorbidity burden (low, moderate, high), malignancy type (solid, hematologic), active malignancy (yes, no), metastatic disease (yes, no), nonelective admission (yes, no), age (continuous), sex (male, female), and hospital teaching status (urban teaching, non-teaching). Reference categories were as follows: comorbidity burden (low), malignancy type (solid), active malignancy (no), metastatic disease (no), nonelective admission (no), sex (male), and hospital teaching status (non-teaching). "Primary analysis" refers to the main multivariable logistic regression model examining the association between high comorbidity burden (ECI ≥5) versus low comorbidity burden (ECI 0-2) and in-hospital mortality in the full study population after applying all inclusion and exclusion criteria. ECI: Elixhauser Comorbidity Index.

We performed several sensitivity analyses to test whether our findings held under different conditions. When we treated the comorbidity index as a continuous measure, each one-point increase was associated with a 19 percent increase in the odds of death. When we limited the analysis to patients with solid tumors that had not spread, the association between high comorbidity burden and death remained. After propensity score matching to balance characteristics between high- and low-burden groups, the effect size was similar. When we separated patients by cancer type, high comorbidity burden remained a predictor in both solid tumors and hematologic malignancies. When we separated patients by whether the cancer was active, high comorbidity burden remained a predictor in both groups (Figure 4).

Secondary outcomes also differed across comorbidity groups. Discharge to a skilled nursing facility occurred in 18.4% (n=2,176) of patients overall, with rates increasing from 12.3% (n=422) in the low-burden group to 26.8% (n=1,220) in the high-burden group. Median length of stay was three days overall and increased across groups. Total hospital charges also increased with comorbidity burden.

## Discussion

In this nationally representative study of patients undergoing TAVR who also had cancer, we found that a high burden of other medical conditions was common, affecting 38.6% of the group. In-hospital mortality rose with increasing comorbidity burden, from 1.1% among those with few other conditions to 5.4% among those with many. This relationship held after accounting for age, sex, cancer type, whether the cancer was active, whether it had spread, and hospital factors. Patients with a high comorbidity burden had four times the odds of dying in the hospital compared to those with a low burden [5,9].

Cancer characteristics also mattered. Patients with blood cancers had higher odds of death than those with solid tumors. Those whose cancer was active had higher odds than those whose cancer was in remission. Those whose cancer had spread had higher odds than those without spread. Even after accounting for these cancer-related factors, comorbidity burden remained the strongest predictor of in-hospital death [5,15].

A related phenomenon worth noting is the "obesity paradox" in TAVR, wherein overweight and obese patients often have lower post-procedural mortality than normal-weight patients despite higher complication rates [16]. This counterintuitive finding suggests that metabolic reserve or other protective factors may modify TAVR outcomes in ways that interact with comorbidity burden. Whether this paradox holds in cancer patients undergoing TAVR remains unknown and warrants further investigation.

Our findings build on earlier work in this area. Landes and colleagues reported that transcatheter aortic valve replacement can be performed safely in patients with cancer, with short-term mortality around 4 percent [17]. Aikawa and colleagues found that patients with active cancer had higher in-hospital death rates than those without active cancer [18]. However, those studies did not fully account for the broader burden of other illnesses that often accompany cancer. Our results suggest that previous estimates of the risk tied to cancer itself may have been too high because they did not adequately separate the effect of cancer from the effect of other conditions that are common in this group [19].

A key contribution of this study is the detailed breakdown of outcomes by cancer type and by whether the cancer was active. Patients with blood cancers had a 62 percent higher odds of death than those with solid tumors. Several factors may explain this correlation. Blood cancers often are characterized by weakened immune systems, low blood counts, and higher infection risk, all of which could increase the risk of a procedure. In our data, patients with blood cancers were also more likely to be receiving active treatment at the time of the procedure. Active cancer itself was associated with an 89 percent higher odds of death, even after accounting for whether the cancer had spread and for the burden of other illnesses. This finding highlights the importance of knowing whether a patient is in active treatment or has a remote history of cancer [20].

Our results have several implications for clinical practice. When assessing risk before transcatheter aortic valve replacement, the overall burden of other medical conditions should be measured with a tool such as the Elixhauser index rather than focusing only on whether cancer is present. The fourfold higher mortality among patients with a high burden of other conditions should be part of discussions with patients and families. For patients with many other illnesses and a limited outlook from their cancer, alternatives to the procedure may be worth considering. For patients with a high comorbidity burden, efforts to improve nutrition, assess frailty, and coordinate care between heart and cancer specialists before the procedure may be beneficial. The higher risk seen with blood cancers and active cancer suggests that these patients may need closer monitoring, attention to infection prevention, and careful timing of the procedure relative to their cancer treatments [21].

This study has strengths. The National Inpatient Sample allowed us to examine a large group of patients from across the country, making the findings broadly applicable. We used a careful approach to measuring comorbidity by leaving out cancer-related diagnoses to avoid bias. We provided detailed information on cancer type and whether the cancer was active, which has been missing from much of the earlier literature. We performed several sensitivity analyses, and the results were consistent [22].

Several limitations should be considered. The National Inpatient Sample relies on diagnosis and procedure codes, which can contain errors. Other studies have shown that codes for TAVR are generally accurate. We lack detailed information on cancer stage beyond whether it had spread, on the specific chemotherapy drugs used, and on the timing of cancer treatment relative to the procedure. We also lack data on frailty and detailed heart function. Our definition of active cancer based on treatment codes may miss some patients whose cancer is active but who are not receiving treatment during the hospital stay [21,23]. The database captures only outcomes during the hospital stay, so we cannot report on survival after discharge or on quality of life. This is especially important for patients with advanced cancer, where the long-term benefit of the procedure may be limited. The database tracks hospital stays rather than individual patients, though this is less of a concern for a procedure that is usually performed only once [12,22]. Our findings apply to patients who were selected for the procedure and may not apply to those who were not. Despite our efforts to adjust for many factors, there may still be unmeasured differences between groups, such as frailty or patient preferences, that could influence both who receives the procedure and how they fare.

Future studies should confirm these findings using clinical registries that contain more detailed information on cancer stage, treatment timing, and longer term outcomes. Researchers should examine whether specific types of chemotherapy or radiation affect outcomes after transcatheter aortic valve replacement. Studies that track patients beyond the hospital stay to capture survival, cancer progression, and quality of life are needed [24]. Better risk scores that combine comorbidity burden, cancer type, and cancer activity with traditional heart risk factors would help guide decisions. Prospective studies are needed to determine the best timing of TAVR in relation to cancer treatment and to define the role of teams that include both heart specialists and cancer specialists in caring for this group [18,25].

## Conclusions

Among patients undergoing TAVR with coexisting malignancy, higher comorbidity burden is associated with a fourfold increase in hospital mortality. This relationship holds regardless of cancer type or whether the cancer is active. What sets this study apart is a more nuanced approach than simply classifying cancer as present or absent. We separately examined overall comorbidity burden, the distinction between solid and hematologic malignancies, and whether the cancer was actively being treated. Each of these factors contributed independently to patient outcomes. Our finding that the burden of other medical conditions predicted mortality more strongly than the mere presence of cancer suggests that earlier research may have overestimated the risk attributable to malignancy alone.

For clinical practice, physicians should look beyond whether a patient simply has cancer. A complete picture of other medical conditions matters just as much as the cancer itself. Blood cancers, active disease, and metastatic spread each add further risk. Heart teams should work closely with cancer specialists to decide who is best suited for the procedure and when to perform it. Some patients with many other illnesses and aggressive cancers may be better served by alternative options. Future research must track patients over longer periods and collect richer information on cancer stage, treatment timing, and overall health to build better decision tools. This work is essential for an aging population where heart disease and cancer increasingly overlap.
